# Understanding dropout stabilization and the factors affecting the return to primary school: a qualitative study

**DOI:** 10.25122/jml-2022-0022

**Published:** 2023-07

**Authors:** Ayoub Eslamian, Maliheh Arshi, Siyamak Tahmasebi, Fardin Alipour, Hassan Rafiey, Gelavizh Karimijavan

**Affiliations:** 1 Department of Social Work, University of Social Welfare and Rehabilitation Sciences, Tehran, Iran; 2 Department of Preschool Education, University of Social Welfare and Rehabilitation Sciences, Tehran, Iran; 3 Department of Social Welfare, Social Welfare Management Research Center, University of Social Welfare and Rehabilitation Sciences, Tehran, Iran; 4 Department of Speech Therapy, School of Rehabilitation Sciences, Tabriz University of Medical Sciences, Tabriz, Iran

**Keywords:** dropout, dropout stabilization, returning to school, primary school

## Abstract

This study aimed to understand dropout stabilization and the factors affecting the return to primary school using a qualitative approach. Data were collected from 47 semi-structured interviews with dropouts, their parents, children who returned to school, social facilitators, school teachers, local community religious leaders, and education activists. Following Graneheim and Lundman's method, thematic data analysis revealed two main categories: “dropout stabilization facilitators” and “dropout stabilization inhibitors”, with 10 subcategories. Dropout stabilization facilitators included the 7 subcategories of perceiving academic decline, inhibitory effects of shame, barriers to re-enrollment, relief from educational pressures and norms, the transformed value system, family satisfaction, and the inefficiency of the formal care system. On the other hand, dropout stabilization inhibitors included the three subcategories of sensitivity to the informal care system, financial incentives, and intensive and flexible training. Some events after dropping out of school resulted in stabilized and definite dropouts. Children at primary schools do not have a clear understanding of the importance of achievements and the effects of education, perceiving dropout as an escape from educational norms and associated hardships. Transitioning into adult roles, such as marriage, motherhood, and assuming responsibilities for siblings, often leads individuals to prioritize other aspects of life over educational achievements. Barriers, such as age limits for being admitted to schools and lack of mechanisms for compensating tuition fees, lead to family dissatisfaction and further reinforce the decision to withdraw their children from school. Factors such as timely actions and follow-ups by schools, financial incentives, and brief interventions provided by the informal network are likely to prevent students from dropping out of school.

## INTRODUCTION

Leaving school during primary education is both an individual and a social issue with negative long-term effects on the social development and economic growth of a society [1]. School dropout is not a problem facing just a specific country. Global data in 2013 indicated that 124 million children either never attended or left school, most of whom were girls living in rural areas [2]. In Honduras and Nicaragua in Central America, school dropout rates in primary education were 42 and 43%, respectively [3]. Despite a primary school enrollment rate of 95% in Brazil, only 59% of the students complete the eighth grade [4]. Numerous statistics have been released on dropout rates in Iran. According to the statistics provided by the latest population and housing census in 2016, there were 315,000 school dropouts aged 6 to 14 in Iran [5]. According to the statistical yearbook of the Iranian Ministry of Education in 2016 [6], 747,000 children dropped out of school until that date. However, the dropout rate varies greatly across the 32 provinces of Iran. According to both statistics above, the Sistan and Balochistan province was the most deprived in Iran. Although this province accounts for less than 4% of the total population of Iran [7], it had the highest school dropout rate in the country, with 49,000 and 115,000 individuals, respectively [5, 6].

The dropout rate is an important indicator of the educational status of countries, being helpful in identifying recent and future problems [4]. The study by Mowen and Brent [8] showed how dropping out of school significantly increased juvenile arrest and detention. Cohen and Soto’s study (2007) established a significant positive relationship between schooling (education) and income per capita. In other studies, increased educational coverage of children and adolescents resulted in a reduced mortality rate of adolescents [9] and improved their health status [10-12].

There are many reasons for dropping out of school, including poor student performance, parents’ wrong attitudes and beliefs, living in a single-parent family [13-15], hating school, having conflicts with teachers as well as school policies [16, 17], grasping job opportunities for one’s low-income family [18], school transportation costs, school uniforms, and tuition fees [19-21]. While many studies have primarily focused on investigating the dropout process until the point of dropout, some authors, such as Aarkrog *et al*. [22], focused on the processes leading to school dropout. Accordingly, instead of focusing on the exact moment of the dropout, they focused on the actions and complexities of this process. Finn's (1989) participation-identification model explains the dropout process and refers to the role of children’s reduced sense of belonging arising from their non-participation as well as the reduced academic performance in their decision to drop out [23]. In another model explaining the dropout process, Finn discussed frustrating self-esteem, where poor educational performance leads individuals to question their abilities [24] and behavioral issues, leading to dropping out of school [23].

As already mentioned, most studies in this field progressed to the point of dropping out of school, yet they did not address the issue of dropout stabilization. Against this backdrop, this study aims to answer the question: “Is the identification of dropout facilitators and inhibitors in primary education effective?”. This study was conducted in the Sistan and Baluchestan province with the highest dropout rate in Iran.

## MATERIAL AND METHODS

This qualitative study used the content analysis method to explore dropout stabilization and the factors influencing the return to primary school. The decision to utilize a qualitative approach was motivated by the novelty of the topic and the limited knowledge surrounding it. Data were collected through semi-structured interviews, with each interview lasting between 40 to 70 minutes. Open-ended questions served as the starting point for the interviews, such as “How did you make sure you would never go back to school?”, “What do you think prevented you from returning to school?” and “What could make you go back to school?”.

In addition, parents, teachers, and other participants were asked some questions about the reasons for not returning to school and stabilizing children’s dropouts. Follow-up questions like “Can you explain more about this?” and “Do you mean …?” were used to encourage elaboration.

Data analysis followed the approach proposed by Graneheim and Lundman [24] to simultaneously analyze, process, and encode the data. Accordingly, the concepts were developed using the constant comparative method. The coding and analysis process began early in the data collection stage, with concepts developed through comparisons between subcategories [25]. The aim was to achieve high intra-category and inter-category homogeneity [24]. The process continued until data saturation was reached [25]. This article is part of a larger study investigating the process of primary school dropout.

### Trustworthiness

Several strategies were employed to ensure trustworthiness, including allowing participants to familiarize themselves with the topic, checking the data with interviewees, and conducting review and coding [26] by co-authors who were social work doctoral candidates with expertise in the subject matter [27].

### Settings and participants

The study was conducted in the Sistan and Baluchestan province, which represents 10.98% of Iran's total area and has a population of 2,775,014 people.

The province comprises 48.5% urban and 51.5% rural areas, with approximately 32% of urban areas classified as informal settlements. This province has the highest percentage of the population below the poverty line in urban areas of the country. It is characterized by significantly elevated poverty rates, specifically in the sectors of finance, banking, energy, education, housing, and welfare facilities. Moreover, it ranks among the most economically deprived provinces in terms of healthcare. The province also has the lowest literacy rate in the country (79.37%). According to the 2016 Population Census, it has the highest educational deprivation rate (8.64%) in children aged 6 to 14.

The data for this study was collected through purposive sampling, involving 47 interviews with a diverse group of participants. Among them were 23 dropouts, 12 girls and 11 boys, and 7 children under 15 who had successfully returned to school (2 girls and 5 boys). In addition, 5 parents, 2 primary school teachers, 2 primary school principals, 3 education specialists, 3 social facilitators, and 2 local religious leaders were interviewed. The collected data were then analyzed using the constant comparative method, as outlined in another study [24]. This method allows for systematic comparison and identification of patterns within the data, contributing to a comprehensive understanding of the research topic.

## RESULTS

### Demographic characteristics of participants

Out of 30 study participants, 16 were girls, 8 were in the first grade of primary school, 23 were dropouts, and 25 had an illiterate father (Table 1).

**Table 1:** Demographic characteristics of participants

Participant characteristics		N (%)
Gender	Boy	14(46.6)
	Girl	16(53.4)
Grade of school dropout	1^th^	8(26.6)
	2^th^	7(23.3)
	3^th^	7(23.3)
	4^th^	3(10)
	5^th^	2(.06)
	6^th^	2(.06)
Educational status	Drop out	23(76.6)
	Returned to school	7(23.4)
Father's educational level	Illiterate	25(83.3)
	Elementary level	2(.066)
	Religious literacy	3(10)
Mother's educational level	Illiterate	28(93.3)
	Elementary level	2(.066)
Marital status	Married	3(10)
	Single	27(90)

During the data analysis process from 1473 obtained codes, the two main categories of “dropout stabilization facilitators” and “dropout stabilization inhibitors,” as well as 10 subcategories, were identified. Dropout stabilization facilitators included the 7 subcategories of perceiving academic decline, inhibitory effects of shame, barriers to re-enrollment, relief from educational pressures and norms, transformed value system, family satisfaction, and inefficiency of the formal care system. In addition, dropout stabilization inhibitors included the three subcategories of sensitivity to the informal care system, financial incentives, and intensive and flexible training (Table 2).

**Table 2:** Examples of the initial codes

Category	Subcategory	Initial codes
Inefficiency of the care system	Poor follow-up of the school	Procrastination in pursuing dropout cases Making a phone call but doing nothing else Failure to visit dropouts’ houses and interfere with their family affairs Lack of mediation and resolution of tensions
	Legal intervention gap	Lack of a legal mechanism for identifying school dropouts Failure to request a list of school dropouts by the judiciary system Failure to punish parents preventing their children from continuing education
	Poor supporting system	Lack of connection between the school and supporting organizations Indifference of child support organizations to children dropout Lack of family access to tuition compensation packages Lack of targeted programs for assigning incentive packages to children returning to school

### Dropout stabilization facilitators

In this section, particular attention is given to the factors that contribute to the stabilization of dropout, like circumstances that result in the permanent discontinuation of education. These influential factors are elaborated upon in this section, with detailed explanations in Table 3.

**Table 3:** Categories produced from data extraction

Main category	Category	Subcategory
Dropout stabilization facilitators	Inefficiency of the formal care system	Poor school attendance Legal intervention gap Poor supporting system
	Perceiving academic decline	Feeling unable to relearn Forgetting course content Loss of educational competitiveness
	Inhibitory effects of shame	Shame of being with young children Shame of the effects of puberty
	Barriers to re-enrollment	Age limits for re-enrollment Priority to enrolment of children at the normal school age Reluctance to enroll married individuals
	Relief from educational pressures and norms	Relief from academic stress Relief from difficulties of doing homework Relief from clothing norms Relief from difficulties of timely attendance
	Transformed value system	Changes in resources give a sense of being valuable Changes in concerns Changes in roles
	Family satisfaction	Reduced educational costs Children’s financial achievements Children’s cooperation in household affairs
Dropout stabilization inhibitors	Sensitivity of the informal care system	Interventions by relatives Support from informal institutions
	Financial incentives	Nutrition at school Covering tuition fees Incentive packages for families
	Intensive and flexible training	Aggregation of educational years Limited training to a few days a week

### Inefficiency of the care system

One of the facilitators of dropping out of school, being a serious obstacle to returning to school, is the inefficiency of the care system in adopting timely measures to help children return to school. In many cases, brief interventions, such as “mediation in resolving family and school conflicts,” lead to school return, being lost in the absence of a school-based social work system. The lack of adequate support systems to address the educational needs of parents and the inefficiency of legal interventions, despite the legal consequences of preventing children from receiving education during their childhood, pose significant barriers to the return to school and contribute to dropout stabilization. Moreover, the inability to activate the local care system, especially the capacity of the highly esteemed religious leaders among people, has been exacerbating this issue (Table 2).

*“We met with the department of education several times, but they were not willing to cooperate with us. We can convince families, but they do not give us information on dropouts”*, a local religious leader stated.

Some children believed that a simple mediation and relieving family tensions with the school could prevent their decision to drop out. The lack of a social work unit at school was one of the problems mentioned in this regard. In addition, the lack of follow-ups and passivity of the school in this regard was mentioned as the other problems.

*“I did not enroll at the beginning of the year, yet they just called me only once. If someone had talked to my father, they could have persuaded him to send me back to school. However, there was no one to do that, and no follow-up was carried out”*, a 12-year-old boy stated.

*“After my mother’s conflict with the school principal, I stopped going to school. Afterwards, no one did anything in this regard, so I could not go back to school”*, a 10-year-old girl said.

The absence of an authority in charge of dropouts at the level of the Ministry of Education could have been the reason for the inefficiency of school care.

*“Here, the department of education does not consider itself responsible for the children having dropped out of school. They believe that as long as a child is present at school, it is within the responsibility of their department. Besides, it is the responsibility of other supporting organizations to care about dropouts. This is the reason why they do not see themselves responsible for doing anything in this regard”*, an educational activist stated.

The lack of access to all kinds of basic support, especially among poor and low-income families, as well as the indifference of the judiciary system towards parents preventing their children from continuing education are other possible reasons for not returning to school among dropouts. Some children could be prevented from staying at home with minor medical interventions.

*“I suffered from anemia. After leaving school a few times, I felt dizzy every time I went back to school. My family felt tired, so they asked me to stay home and not go back to school”*, an 8-year-old female dropout said.

The lack of sensitivity demonstrated by the legal system and the absence of follow-up measures were identified as additional facilitators of dropout.

*“Despite regulations on compulsory education and criminal nature of preventing children from continuing education, we do not see any practical measures adopted in this regard. Besides, supporting organizations do not prioritize children’s right to receive education. Some children need make-up classes to refrain from academic failure”*, an educational activist stated.

One of the school dropouts (a 10-year-old child) confirmed this by stating:

*“No one followed up on me from the school. They just called once, yet no one pursued my affairs from the school. If they had come to my house and talked to my father, they could have convinced him to send me back to school”*.

The lack of communication between supporting organizations to identify poor children at school and the lack of timely incentive packages and financial support were other examples of formal care system inefficiency, which caused many children to drop out of school due to poverty.

### Perceiving academic decline

Feeling unable to learn again, forgetting course contents, and losing educational competitiveness, especially after dropping out of school, were among the major factors making children reluctant to return to school.

*“I no longer thought I would learn anything again. I thought the lessons had become too difficult for me, and I lost my mental power to learn anything”*, an 11-year-old girl, 3 years after dropping out of school, stated.

Another important factor, in addition to feeling unable to learn again, was forgetting the course content, as mentioned by the dropouts.

*“I forgot everything I knew. It sounds as if I have never been to school and have not studied at all”*, a 14-year-old boy, 4 years after dropping out of school, said.

### Inhibitory effects of shame

The feeling of shame emerged as a significant barrier preventing dropouts from returning to primary schools. The children referred to the “shame of being with younger children” and the “shame of the effects of puberty” to describe obstacles to their return to school. Many children felt embarrassed to be notorious in the class, for they were physically different from other children. This feeling caused them to drop out of school again if they returned.

*“I went back to school for a day or two, but some small kids over there were not the same size and height as me. Thus, I felt embarrassed to be there”*, an 11-year-old girl, 3 years after dropping out of school, said.

On this issue, one of the primary school teachers said:

*“Some children, especially girls reaching puberty and having periods, are embarrassed to go back to school. They think they no longer belong to the class and this age group”*.

In fact, this experience was associated with losing a network of former friends, which diminished the dropouts’ motivation for returning to school.

*“All my friends had left that school, so I had no friends over there. In addition, I was much older than anyone else, with no one in the class willing to make friends with me”*, a 10-year-old boy said.

### Barriers to re-enrollment

“Age limits for re-enrollment”, “the prioritization of enrolling children of the typical school age”, and “reluctance to enroll married children” often prevented dropouts from returning to the same school.

A teacher pointed to the age limit for returning to school as follows:

*“Most schools are unwilling to enroll children who have left school. Besides, there is an age limit for returning to school. For example, they must not be over 9 to attend the first grade. These children must go to classes held by the Literacy Movement Organization of Iran, about which they may not know anything, or they may not be willing to study with adults”*.

In some cases, there is no legal ban on returning to school, but schools prefer to enroll school-age children.

*“When two people come to enroll at the school, priority is given to enrolling our students rather than the older ones who may be married, with other kinds of troubles,”* a primary school teacher stated.

### Experience of relief

Leaving school, although painful for children, is somehow associated with the experience of relief from academic stress, difficulties in doing homework, clothing norms, and difficulties in timely attendance at school.

*“I got rid of going to school every day and the troubles of the school. I used to walk a long distance every day. Now I’m at home and feel comfortable. That’s because I no longer have to study”*, an 8-year-old girl, 9 months after dropping out of school, said.

A 9-year-old boy pointed to the troubles of going to school and the feeling of relief from that after dropping out of school as follows:

*“We had to go to school at 7 a.m. and walk about one hour every day. If we arrived late, we would be beaten. I feel well now, and I do not have to work hard”*.

The children’s lack of accurate understanding of the importance of education and its achievements at an early age caused them to feel relief from academic stress and troubles as a pleasant experience. In this regard, a primary school principal said:

*“I think every child is happy for not going to school and playing on the street. Many children here have no responsibilities until they go to school, so they just play and play”*.

Some families had the feeling of liberation as well. In addition, most families felt relieved because they did not have to be constantly involved in enrolling their children, accompanying them to school, helping them with homework, etc.

*“Families welcome their children’s dropping out of school. A father used to tell me that he was happy that he no longer had to accompany his child to school every morning. Some people are happy that they do not have to practice dictation or math with their children at night”*, a social facilitator stated.

### Transformed value system

Dropouts gradually enter a new world with changes in resources. Accordingly, they try to get a sense of being valuable, with concerns and roles completely different from those of school.

In our study, they no longer enjoyed being valuable after obtaining a high score and did not feel proud of other achievements. In fact, they thought about trying other things. These children no longer introduced themselves with a sense of student identity. They also considered themselves adults seeking and receiving feelings of worth and satisfaction from other sources in terms of earning money, marriage, and motherhood.

*“I no longer think and care about scores. All I can do now is learn how to work more quickly and be able to stand on my own feet. All my friends have savings and help their families”*, a 13-year-old boy said.

*“I think about my childhood every day and night and believe that going back to school is a waste of time. Education is for children, yet when you get married, you no longer think about school at all”*, a 14-year-old girl stated.

A school principal pointed to children’s being accepted in the adult community as follows:

*“Children are happy to work with adults and to be accepted in their community. They feel proud and consider it an experience they may never feel at school”*.

### Family satisfaction

Reduced educational costs, children’s financial achievements, and their cooperation in household affairs made families satisfied with their children’s dropping out of school. Thus, they did not attempt to persuade their children to return to school.

*“Children’s education is an investment for the future. However, for families involved with their present needs, children’s work is more satisfying, for it helps their families’ economy”*, a primary school teacher said.

Caring for other children or sick family members was another responsibility that children’s education prevented them from fulfilling.

*“Here, women give birth until old age, with the birth rate being much higher than the national average. Accordingly, most girls are involved in caring for their new siblings. These children are not confused after leaving school and have clear responsibilities welcomed by their families. Besides, there is always a lot of housework for dropouts to do”*, an educational activist stated.

Dropping out of school caused the children to be free to play all these roles, being accompanied by family satisfaction. This feeling of satisfaction prevented the family from seeking or demanding their children return to school. Child labor was one of the main reasons for parental satisfaction in this regard.

*“My father encouraged me to assist him with the farm activities every day. He used to be alone, yet we are now working together”*, a 14-year-old boy said.

### Dropout stabilization inhibitors

According to the findings, some factors stabilized the return to school and prevented children from dropping out. These factors included informal care, financial incentive system, as well as intensive and flexible training, being the three main categories in this respect. In this section, these categories are discussed in detail.

### Informal care

In the absence of a formal care system, it would be possible to return to school if there was access to informal care. Interventions made by relatives were considered part of this case.

*“I would not go back to school if my uncle did not insist and follow up on it”*, a 13-year-old boy who returned to school said.

*“No one interfered here; no one interferes in family decisions at all. Relatives themselves prevent their children from studying, yet those who agree with education do not interfere in that”*, a 13-year-old girl stated.

In many cases, follow-up and support from informal institutions prevented dropout stabilization. For example, philanthropists and mosques helped and interfered to persuade parents to forgive their children, which were among such measures.

*“Because of a mistake a child made, the family prevented her from returning to school, and after we followed up on it and talked to the family, the father agreed to send his daughter back to school,”* a local religious leader said.

One of the successful measures to help children return to school was the initiative of a charity-sponsored school on the outskirts of Zahedan city, providing breakfast to children. A number of children returned to school using this plan. The principal of this school pointed out:

*“There are a lot of poor people here, and even this simple breakfast encourages people to send their children back to school”*.

### Financial incentives

One of the incentives for making children return to school, even after a long time, was financial incentives. The experience of some of the teachers and the children returning to school confirmed this.

*“Many parents made their children return to school having realized that if their child returned, they would receive tuition fees and some financial subsidies. I wish this policy was always in place and was not just a temporary plan”*, a social facilitator stated.

### Intensive and flexible training

We already mentioned that a significant barrier to returning to school is feeling inadequate in learning the course material. The action of a number of non-profit schools showed that there would be the possibility of returning to school if extensive make-up training courses were provided.

*“Here, we train dropouts on weekends and often run several classes together. According to educational regulations in Iran, if they pass intended exams for a given grade, they will be allowed to go to a higher grade, regardless of their last grade, when attending school. For instance, we had children who studied up to grade two. We taught them lessons of two or even three grades (if they were smart enough) simultaneously. For instance, if they passed the exams for the fifth grade, they would be authorized to enter grade six. This project was welcomed by some families”*, a primary school principal stated.

*“When the principal of this non-profit school told my mother that I did not have to go to school every day, she agreed that I would study for a day or two”*, a 10-year-old girl who returned to school said.

## DISCUSSION

This study aimed to investigate the reasons for stabilizing dropout as well as barriers to returning to school in the Sistan and Balochistan province. The results of this study identified some factors effective in leading to dropout stabilization and children’s failure to return to school.

The result of this study showed that the two main categories of “dropout stabilization facilitators” and “dropout stabilization inhibitors” and 10 subcategories were identified. Dropout stabilization facilitators included the 7 subcategories of perceiving academic decline, inhibitory effects of shame, barriers to re-enrollment, relief from educational pressures and norms, transformed value system, family satisfaction, and inefficiency of the formal care system. In addition, dropout stabilization inhibitors included the three subcategories of sensitivity to the informal care system, financial incentives, as well as intensive and flexible training.

The feeling of relief from difficulties and norms of education was a factor that reduced the children’s motivation for returning to school. In line with the findings of the present study, unpleasant experiences at school, such as strict school policies [28], inappropriate conditions and the amount of control at school [29], as well as poor quality of education [20] were among the causes of dropping out of school. Accordingly, leaving school with such an unpleasant experience seemed to cause a sense of liberation. In addition, it was a source of satisfaction for children and prevented them from being willing to return to school.

Losing the opportunity to become a classmate with former friends, the shame of being with younger children, and the possibility of being rejected and ridiculed by this younger group were among the other obstacles contributing to dropout stabilization, having been closely related to an increase in the children’s distance from the school. Finn’s theoretical explanation for the decrease in children’s sense of belonging as a factor for dropping out of school was in line with this situation. Failure to participate in school programs harms a child’s sense of belonging [23]. The results of this study showed that the loss of a friendship network was the source of the lack of belonging, i.e., the feeling of isolation and loneliness.

Another factor stabilizing children’s dropout was their perceptions of their inability to learn again after dropping out. Accordingly, they assumed they had forgotten lessons and lost the mental capacity for relearning. Thus, they felt unable to compete with other children. These results remind us of Finn’s frustration-self-esteem model, according to which poor academic performance results in questioning children’s abilities [24], being considered a path for dropping out of school.

The family’s role in stabilizing their children’s dropout was significant, especially at an early age when the children often did not have much independence to make decisions. The reduction in tuition fees, the children’s financial achievements, and their role in household chores, such as caring for sick family members and younger siblings, was considered a blessing for the family and a major barrier to the children’s return to school. Other studies mentioned the impact of unemployment and family poverty [15, 18, 30, 31] as well as the lack of free education [32] and family support (being pregnant or looking for a job to support the family) [14-16] on dropping out of school by children. In other words, these studies referred to the family’s important role in causing and stabilizing dropout. The sense of family satisfaction is transmitted to the children and causes a sense of worthiness in them. For example, grasping early job opportunities in low-income families [29, 18] can lead to drop out. Accordingly, the results of this study showed that such valuable achievements by dropouts could create hope for their families.

Among barriers to stabilizing dropout or incentives for returning to school, one can consider financial incentives, school meals, and tuition fees. The findings of a prior study [33] are consistent with the results of the present study, confirming the influence of the lack of free education on dropout rates [33]. In addition, it showed that family support could increase the likelihood of returning to school. The results of this study were consistent with those of Laxmaiah *et al*. [34] and Gallenbacher (2018) [35], which reported that the meal plan significantly prevented children from dropping out of school. On the other hand, Schultz’s study [36] reported that paying grants to poor rural mothers significantly increased student enrollment rates. The need for such support seems highly essential, especially in the Sistan and Baluchestan province, the most deprived province in Iran. Having a poverty rate of 43.5%, this province has the highest poverty rate in the country. Furthermore, it is noteworthy that approximately 32% of the population residing in the urban areas of the province live in informal settlements [13]. This study showed that poverty can be closely associated with dropout. The findings from a study conducted by De Witte *et al*. [19] revealed that children from families belonging to low socioeconomic classes, particularly those with low social capital, geographically marginalized backgrounds, and unemployed parents, were more susceptible to dropout. Obstacles to stabilizing dropout included follow-ups and interventions by schools or other institutions. The results of this section were similar to the effectiveness of the Check Connect program in the study by Goulet *et al*. [32], which was an effective program in reducing dropout.

Moreover, the study by Vitaro *et al*. [37] showed that behavioral interventions led to a reduction in dropouts. The present study showed that intervention measures, family follow-ups, as well as legal and welfare protections were causative factors for children returning to school. Such support could be provided by informal institutions, such as non-governmental bodies or relatives.

Sud conducted a study [38] in India on informal and charity-sponsored schools to examine the impact of intensive and informal education. Accordingly, the results of his study confirmed the success of those schools, especially in underprivileged and marginalized areas.

This study has some strengths, such as the inclusion of participants from diverse groups, including dropout children, parents, and experts. A limitation of this study is its qualitative design, which may affect the accuracy of participants' recollection of past events, and due to the social utility, the responses could have been over realistic, and care should be taken to generalize these results.

## CONCLUSION

The results of this study indicated that individuals, families, school-related care systems, and social factors were effective in stabilizing dropout and creating the opportunity for returning to school. Long-time detachment from a previous situation leads to getting immersed in new roles, being involved with new concerns, and feeling committed to doing new tasks, which are likely to reduce one’s desire and possibility for returning to school. According to the results of this study, the impact of timely interventions, financial incentives, and brief interventions by relatives, in some cases, prevented dropouts from being stabilized. In general, the results of this study showed that there was an opportunity for returning to school after dropping out of it. In the end, it is worth noting that these results can inspire social efforts in the field of education.
